# Mother of "a Living or Viable Child," Aged Forty-Eight, or over?

**Published:** 1917-03-10

**Authors:** Fred. J. Smith

**Affiliations:** 138 Harley Street, W.


### THE EDITOR'S LETTER-BOX.

### Mother of "a living or viable child," aged forty-eight, or over?

*To the Editor of* THE HOSPITAL.

SIR,—I wish to establish, if possible, as a fact whether there is or is not a case of "a woman aged forty-eight or over having a living or viable child," recorded *on evidence other than the mere ipse dixit* of the woman as to her age. Might I enlist the assistance of your readers to enable me to settle the point by reporting to me any cases within their knowledge?

I am, of course, aware that there are a good many reported on the evidence of the mere statement of the woman that she was forty-eight or older; but as the Registration of Birth Act is now over fifty years old it should be possible to get the date of parturition and the date of the mother's birth both officially recorded, and it is cases of this nature I am anxious to obtain for a forthcoming edition of "Taylor's Medical Jurisprudence."—Yours truly. FRED. J. SMITH.

138 Harley Street, W.

